# Wide-field mosaics of the corneal subbasal nerve plexus in Parkinson’s disease using in vivo confocal microscopy

**DOI:** 10.1038/s41597-021-01087-3

**Published:** 2021-11-26

**Authors:** Reza A. Badian, Stephan Allgeier, Fabio Scarpa, Mattias Andréasson, Andreas Bartschat, Ralf Mikut, Alessia Colonna, Marco Bellisario, Tor Paaske Utheim, Bernd Köhler, Per Svenningsson, Neil Lagali

**Affiliations:** 1 Unit of Regenerative Medicine, Department of Medical Biochemistry, Oslo University Hospital, Oslo, Norway; 2 Institute for Automation and Applied Informatics, Karlsruhe Institute of Technology (KIT), Karlsruhe, Germany; 3 Department of Information Engineering, University of Padua, Padua, Italy; 4 Center for Neurology, Academic Specialist Center, Stockholm, Sweden; 5 Department of Neurology, Karolinska University Hospital, Stockholm, Sweden; 6 Department of Clinical Neuroscience, Karolinska Institutet, Stockholm, Sweden; 7 Division of Ophthalmology, Institute for Biomedical and Clinical Sciences, Linköping University, Linköping, Sweden; 8 Department of Ophthalmology, Oslo University Hospital, Oslo, Norway; 9 Department of Ophthalmology, Sørlandet Hospital Arendal, Arendal, Norway

**Keywords:** Predictive markers, Prognostic markers, Parkinson's disease

## Abstract

*In vivo* confocal microscopy (IVCM) is a non-invasive imaging technique facilitating real-time acquisition of images from the live cornea and its layers with high resolution (1–2 µm) and high magnification (600 to 800-fold). IVCM is extensively used to examine the cornea at a cellular level, including the subbasal nerve plexus (SBNP). IVCM of the cornea has thus gained intense interest for probing ophthalmic and systemic diseases affecting peripheral nerves. One of the main drawbacks, however, is the small field of view of IVCM, preventing an overview of SBNP architecture and necessitating subjective image sampling of small areas of the SBNP for analysis. Here, we provide a high-quality dataset of the corneal SBNP reconstructed by automated mosaicking, with an average mosaic image size corresponding to 48 individual IVCM fields of view. The mosaic dataset represents a group of 42 individuals with Parkinson’s disease (PD) with and without concurrent restless leg syndrome. Additionally, mosaics from a control group (n = 13) without PD are also provided, along with clinical data for all included participants.

## Abstract (summary)

**Table:** 

Measurement(s)	Prominent corneal nerve fibers • Dendritic Cell
Technology Type(s)	confocal microscopy
Factor Type(s)	disease status • comorbidity
Sample Characteristic - Organism	Homo sapiens

Machine-accessible metadata file describing the reported data: 10.6084/m9.figshare.16691452

## Background & Summary

Neurological disorders are a significant cause of disability and death worldwide. In the period from 1990 to 2015, the number of deaths from neurological disorders increased by 36.7%, with Parkinson’s disease (PD) constituting the 12^th^ most common condition leading to premature death (1.2%) among all neurological disorders in the overall global burden from neurological disorders in 2015^1^.

PD is a progressive neurodegenerative disorder with its diagnosis based on clinical criteria consisting of a combination of bradykinesia with rigidity and/or rest tremor^2^. Peripheral neuropathy (PN) that occurs in PD can exhibit both small and large fiber involvement^3,4^. Additionally, restless legs syndrome (RLS), a sensorimotor condition, is frequently reported among patients suffering from neurological disorders including PD; therefore occurrence of RLS as comorbidity in PD patients has been the focus of multiple studies^5–8^. As PN is difficult to diagnose, there has been much interest in the development and refinement of non-invasive examination modalities for its potential diagnosis. Increasingly, quantitative measures derived from non-invasive peripheral nerve imaging in the corneal subbasal nerve plexus are being reported as putative indicators of small fiber peripheral neuropathy in conditions such as diabetes mellitus^9–17^. This technique is therefore of interest in the context of PD and RLS.

The cornea is a densely innervated tissue, whose sensory nerve bundles enter the cornea at the mid-stroma in a radial formation from the periphery^18–21^. The stromal branches extend towards the central anterior cornea, penetrate Bowman’s layer, and then individual nerve fiber bundles separate and run parallel to the corneal surface, at the level of the corneal basal epithelium, forming the sub-basal nerve plexus (SBNP) supplying the sensory nerve fibers to the corneal epithelium^18,20,22^. *In vivo* confocal microscopy (IVCM) is a clinical method of non-invasively examining the cornea, providing images with high lateral (1–2 µm) and axial (5–10 µm) resolution, at a magnification of up to 600^23^ to 800 times^24^. IVCM provides excellent images of the SBNP, from which parameters such as corneal nerve fiber length density (CNFL, measured as the sum of the nerve fiber length in mm divided by the corresponding SBNP area in mm^2^), corneal nerve fiber number density (CNFD, the number of distinct nerve fibers (defined in various ways) per mm^2^ of SBNP area), corneal nerve branch density (CNBD, the number of nerve branching points per mm^2^ of SBNP area), and tortuosity (defined in various ways) can be measured^10,16,18,20,25,26^. Only a few studies have used IVCM to examine corneal nerves in PD with some contradictory results. One study included 26 PD patients with varying disease duration and 26 controls. Using IVCM and selecting 4 to 6 single field-of-view images per eye for analysis, reduced CNFD, but increased CNBD and CNFL were found, compared to (healthy) controls^27^. Another IVCM study, including 26 patients with early PD and 22 controls, analyzed 4 to 8 single field-of-view IVCM images of the SBNP per subject. The authors of the study reported that CNFL and CNBD were significantly reduced in PD patients compared to controls, while CNFD reduction was not statistically significant^28^. Another study assessing 15 patients with moderate PD and 15 healthy controls, did not report the number of IVCM images analyzed per eye, but found a significant reduction in CNFL in PD patients relative to controls^29^.

Here we present an IVCM dataset representing a larger number of patients (n = 42) compared to prior IVCM studies of PD. The raw data in this dataset was originally used^30^ to investigate a possible relationship between the presence of RLS in PD and corneal nerve parameters. Here we provide an entire dataset of high quality wide-field mosaics of the corneal SBNP in PD patients (21 with and 21 without RLS) and 13 age-matched controls. We also provide the relevant clinical diagnostic information alongside the SBNP mosaics. The high quality wide-field mosaic images are a unique distinguishing feature in the present dataset, relative to prior IVCM studies. Our dataset represents the largest SBNP image sizes published to date, from any clinical cohort. The use of mosaics avoids the subjective selection of individual fields of view thus providing an objective view of the overall SBNP architecture, enabling accurate analysis of SBNP patterns and exact quantification of SBNP parameters^16,31^. It has previously been shown that not using mosaics of the SBNP but using only small numbers of hand-selected images can lead to very large errors in the values of reported parameters^31^, possibly explaining wide discrepancies in previously reported values of SBNP in PD populations.

Moreover, inflammation is considered as one of the important etiological processes in PD as a neurodegenerative disease^32–34^. The mosaic dataset provided here additionally contains inflammatory cell parameters that can be further analyzed for their relation to the various clinical disease parameters. To the best of our knowledge, no prior study in PD has investigated the inflammatory cells that are clearly visible at the level of the SBNP, although in other conditions these inflammatory cells (such as antigen-presenting dendritic cells) have been shown to be related to the onset of disease^35^.

## Methods

### Study design, participants, inclusion and exclusion criteria

The initial study from which the raw IVCM data was collected^30^ had a cross-sectional design, where participants were enrolled in the period from Spring 2018 to Autumn 2019 at the outpatient clinic at Center for Neurology and Karolinska University Hospital, Stockholm, Sweden. The study encompassed control participants without PD, and PD patients with (PD + RLS) and without RLS (PD-RLS) matched for age and sex. Participants were aged between 50 and 80 years and had to have one eye without history of previous corneal trauma, surgery or ongoing eye drop treatment. Patients fulfilled a diagnosis of clinically probable PD with or without RLS according to established criteria^2,36^. PD + RLS (n = 21), PD − RLS (n = 21) and controls (n = 13) comprised the study. Written informed consent was obtained from all participants and the study was approved by the regional ethical board of Stockholm, Sweden (ref. nr 2018/264-31/2 (2019-03158)). Inclusion and exclusion criteria have been previously described in detail^30^.

### Clinical assessments

Details of the clinical, neurophysiological and biochemical assessments are outlined in our original study report^30^. In short, demographic and disease-specific parameters were obtained by oral interview. Neurological rating scales included modified Hoehn and Yahr staging (mH&Y)^37,38^ and the Utah Early Neuropathy Scale (UENS)^39^. The severity of RLS symptoms was evaluated with the International Restless Legs Scale rating scale (IRLS)^40^ and the sensory suggested immobilization test was performed in the PD + RLS group^41,42^. With regard to electrodiagnostic and quantitative sensory testing, details are described in the original study report^30^.

### *In Vivo* Confocal Microscopy Examination

*In vivo* confocal microscopy of the cornea was performed to visualize the peripheral small fiber morphology of the corneal SBNP. IVCM image acquisition was conducted in both eyes of all participants, or in one eye in cases where the other eye did not meet the inclusion criteria. A single, experienced examiner performed all examinations using a Heidelberg Retinal Tomograph 3 with Rostock Corneal Module, HRT3-RCM (Heidelberg Engineering, Germany), using a built-in fixation light to bring the focus on to the central cornea. A motorized joystick module was used to control and maintain the focal plane at the desired corneal depth, at the SBNP level. The central and paracentral corneal regions were first imaged by translating the microscope field of view manually in a raster pattern, until regions were reached where the curvature of the cornea resulted in oblique images. Subsequently, to image the paracentral regions, the fixation light was moved sequentially in superior, inferior, temporal and nasal direction, with the manual raster scanning process being repeated for each fixation light position. During scanning, the depth of the SBNP was maintained by manually adjusting the depth of focus by small movements on the joystick, in order to capture subtle variations in the plexus and enable a maximal projection of nerves to be made onto a 2D plane, as previously described^31^.

Differing from prior work, however, during the clinical examinations an attempt was made to image as large a wide-field area of the SBNP as possible by periodically pausing the examination to allow the subject and examiner to rest, then resuming the examination, until the examiner judged the imaged area to be of sufficient extent and quality, provided the subject was willing to cooperate with the strategy. The raw image datasets obtained from IVCM examination were then used as input to automated mosaic generation and nerve detection and quantification algorithms (described below).

### Automated mosaic image generation

The process used to assemble SBNP mosaic images from the acquired datasets is identical to the method described in previous studies^16,31^, with one notable exception described below. The mosaic generation process consisted of four consecutive process steps:

1. Removal of non-SBNP images from the dataset,
2. Pairwise, correlation-based image registration (using a decomposition of the images into 12 horizontal rectangular sub-images),
3. Formation and solution of a system of linear equations, yielding position coordinates of the sub-images, and
4. Construction of the mosaic image.

As it is not always possible to avoid the inclusion of non-SBNP images in the acquired datasets, these images were first removed from the processed data. The benefit of this is twofold. Non-SBNP images could negatively influence the contrast of the relevant SBNP image features if included in the mosaic algorithm, and removal of such images reduces mosaic-processing time if excluded early in the processing pipeline. Whereas the exclusion of non-SBNP images had been done manually in the past^31^, a tissue classification algorithm^43^ was used in the initial PD^30^ study to automatically identify and exclude non-SBNP images. The classifier is based on the Bag of Visual Words approach. It uses a trained feature extraction followed by a set of support vector machines, each of which had been trained to separate one characteristic corneal tissue class (epithelium, SBNP, stroma) from any other tissue class. A previous quantitative evaluation of the classifier reported a classification accuracy of over 96% on a manually labeled set of 663 IVCM images^43^.

The image registration step makes use of the phase correlation function, a well-established approach for calculating the relative offset between two images^44^. The phase correlation was calculated for all possible image pairs of a dataset to establish an estimation of the translational alignment between each image pair, but the key step in creating high-quality mosaic images is the decomposition of each image into 12 horizontal slices or sub-images and the calculation of the relative alignment *d*_*ij*_ (relating to the sub-images with indices *i* and *j*)^31,45^. This approach was designed to analyze specifically the characteristic motion-induced image deformation artifacts that arise from the image formation process of the HRT-RCM microscope.

The third step of the mosaic generation process was the deduction of absolute, global position coordinates *p*_*i*_ for the sub-images from the translation vectors *d*_*ij*_ that had been calculated in the registration step. This global alignment process is based on the observation, that the translation vectors effectively estimate the position differences between respective sub-images, ${d}_{ij}={p}_{j}-{p}_{i}$^31,45^. After excluding all sub-image registration results with a correlation value below an empirically predefined threshold, these equations form a system of linear equations. The linear equation system always possesses degrees of freedom that need to be addressed by additional regularization terms: An additional equation ${\lambda }_{1}\;{p}_{0}=0$ complements the otherwise purely difference equations with an absolute reference, and equations ${\lambda }_{2}({p}_{i+1}-{p}_{i})=0$ (limited to pairs (*i*, *i* + 1) of sub-images that belong to the same original image) provide alignment information for sub-images without any accepted registrations; *λ*_1_ and *λ*_2_ are weight factors. The regularized system of linear equations is subsequently solved for the sub-image position coordinates *p*_*i*_.

The final mosaic image construction step was implemented as described previously^31^. Appropriate interpolation between the sub-image positions *p*_*i*_ yields position coordinates for single image rows, and the final mosaic image was then calculated by weighted averaging of overlapping original image data.

### Optimizations of the mosaic image generation process

Runtime considerations were not a priority in the context of the present dataset. However, with regard to potential application in routine clinical practice (and also with regard to a larger number of patients in the future), the mosaic image generation process was reexamined with a particular focus on runtime optimization. The most effective means to reduce runtime is a size reduction of the images in the context of the image registration step, as the calculation time of the correlation function is dependent on the size of the input data. However, reducing the image information used for the correlation function inherently increases the noise level of the correlation function, making it harder to reliably separate correct registration results from incorrect ones. Scale factors of 3 and 2 for full image and sub-image correlations, respectively, have proven to be a good compromise.

### Automated nerve tracing and nerve parameter quantification in SBNP mosaics

The algorithm used for automated nerve fiber tracing in mosaics was described earlier by Guimarães *et al*.^46^. Briefly, this algorithm is based on three main steps: pre-processing, classification and post-processing. The pre-processing aims to improve the visibility of the corneal nerves. To achieve this goal, a Top-Hat morphological filtering was used to equalize the background and to improve the contrast of the image. A bank of log-Gabor filters, each with a different orientation, highlights linear structures and completes the pre-processing. A threshold was then applied to identify pixels corresponding to a nerve. From the selected pixels, morphological and intensity-based features are extracted and used as input for a finer classification based on the support vector machines approach. The final classification consists in a label “nerve” or “other” assigned to each pixel of the image. A binary image was then obtained, in which white pixels correspond to nerves and black pixels correspond to the other.

The resulting binary image contains nerve segments with small gaps between each other, due to noise in the original image. Thus, to improve the nerve tracing, the post-processing step performs morphological operations and traces missing connections^47^. In addition, the algorithm computes all possible connection-paths between segments based on distance, angle, and intensity. The best connection-path is then chosen based on the Dijkstra algorithm^48^.

From the automated nerve tracing, quantitative nerve parameters were extracted. For the current mosaic dataset, the algorithm provided the mosaic corneal nerve fiber length density (mCNFL), defined as the total length of all nerves in the mosaic divided by the mosaic area (black regions excluded) expressed in mm/mm^2^, and the mosaic corneal nerve branching density (mCNBD) defined as the total number of branching points divided by the mosaic area (black region excluded) expressed as the number of branching points per mm^2^.

The inferocentral whorl region of the subbasal plexus normally contains the highest concentration of subbasal nerves, and this region has been analyzed separately in several studies^31,49–51^. Here, subbasal nerve parameters in the whorl region were analyzed by the automated tracing algorithm described above. Whorl corneal nerve fiber length (wCNFL) and whorl corneal nerve branch density (wCNBD) in the whorl region were automatically calculated from automated tracing with respect to four configurations; for nerves within a full circular region centered on the whorl center with either an 800 µm diameter or 400 µm diameter, and for the corresponding superior semi-circular regions (extending from 9 to 3 o’clock) only. Nerve analyses were performed across control, PD − RLS and PD + RLS groups, and again for controls vs. all PD participants.

### Inflammatory cell analysis within the SBNP

Additionally, it is possible to analyze the mosaic dataset for the presence of inflammatory cells/dendritic cells (DCs), and with respect to the different DC subtypes whose morphologic features have been described in an earlier study^35^. Here, two independent experienced observers performed morphological characterization and quantification of the inflammatory DCs present in the SBNP. The two observers were masked to the identity of each mosaic image. Three types of DCs were quantified: mature DCs, immature DCs and globular cells^35^. The DC density values, expressed as cells per mm^2^ of mosaic area for the various subtypes, were averaged across observers and across both eyes for all participants. This data is also provided along with the mosaic dataset.

### Statistical analysis

Statistical analyses of presented data were performed using IBM SPSS statistics for Windows, version 25.0 (IBM Corp., Armonk, N.Y., USA). A two-tailed P value of < 0.05 was considered significant.

## Data Records

Data for the wide field mosaic images of the corneal subbasal plexus that were acquired for PD patients and control participants are provided^52^. The Excel file contains the numeric data and the corresponding mosaic image numbers, and is therefore the ‘key’ to the mosaic image dataset. IVCM mosaic images represent the largest mosaic per eye and are provided in TIFF format, labeled with the subject ID number (01 to 57 – two participants were excluded hence non-consecutive numbering) and the eye (RE for right eye and LE for left eye). Table 1 details the parameters associated with the SBNP mosaic dataset. In addition, we provide in the same file the clinical parameters linked to each study subject (Table 2). Finally, we provide folders for each eye (in ZIP format) containing the raw, non-stitched IVCM images used to create each corresponding mosaic image.

**Table 1:** Study parameters related to the IVCM data obtained and provided in the mosaic dataset.

Parameter	Description
Subject ID	Identification number assigned to each of the subjects in the study cohort (1 to 57)
Image name	File name assigned to each image in the wide-field mosaics dataset
Eye	Image and clinical data corresponding to right eye (RE) or left eye (LE)
Mosaic area	Area of the corneal subbasal nerve plexus represented in the mosaic image (mm^2^). In all cases, regions without image data (black or empty areas) were not included in the area calculation.
mCNFL	Mosaic corneal nerve fiber length density: the total length of all nerves in the mosaic divided by the mosaic area expressed in (mm/mm^2^)
mCNBD	Mosaic corneal nerve branch density; defined as total number of branching points divided by the mosaic area and expressed as the number of branching points per mm^2^ (nr./ mm^2^)
wCNFL	Whorl corneal nerve fiber length density; defined as corneal subbasal nerve fiber length density in the whorl region (in mm/mm^2^), based on automated nerve tracing. wCNFL is provided for 800 µm and 400 µm diameter whorl regions in full and half-circle areas (see Fig. 3). Only values for eyes where the full circle contained image data are included.
wCNBD	Whorl corneal nerve branch density defined as total number of branching points in the whorl region divided by the mosaic area expressed as the number of branching points per mm^2^. wCNBD is provided for the four different definitions of the whorl as for wCNFL.
mDCs	Density of mature dendritic cells in each mosaic, in cells/mm^2^ of mosaic area.
imDCs	Density of immature dendritic cells in each mosaic, in cells/mm^2^ of mosaic area.
GCs	Density of globular cells in each mosaic, in cells/mm^2^ of mosaic area.

**Table 2:** List of clinical and demographic parameters linked to each study subject in the provided data set.

Parameter	Description
Subject ID	Identification number of the subject in the cohort (57 subjects of which 2 were excluded).
Study Group	Controls, PD + RLS, or PD − RLS
Age	Subject age at time of study inclusion (years)
Sex	1 = Male, 0 = Female
Smoking	Smoking status, 1 = yes, 0 = no
Coffee intake	Consumption of coffee (cups/day)
Intake of B12 supplements or multivitamins	1 = yes, 0 = no
PD motor duration	Duration of motor symptoms (years)
RLS duration	Duration of restless legs syndrome (years)
L-dopa duration	Duration of treatment with L-dopa (years)
LEDD	L-dopa equivalent daily dose (mg)
Ongoing L-dopa therapy	1 = yes, 0 = no
mH&Y (stage)	Modified Hoehn and Yahr scale for staging Parkinson’s symptoms and disability
IRLS	International RLS study group rating scale (IRLS): 0–40
UENS	Utah Early Neuropathy Scale. Clinical rating scale for the assessment of peripheral neuropathy.
p-homocysteine	Concentration of homocysteine in plasma (μmol/L)
s-methylmalonic acid	Concentration of methylmalonic acid in serum (μmol/L)
p-pyridoxal-5′-phosphate	Concentration of pyridoxal-5′-phosphate in plasma (nmol/L)
s-ferritin	Concentration of ferritin in serum (μg/L)

Blood parameters were based on fasting whole blood samples acquired using venipuncture. L-dopa: Levodopa.

## Technical Validation

**Fig. 1:**
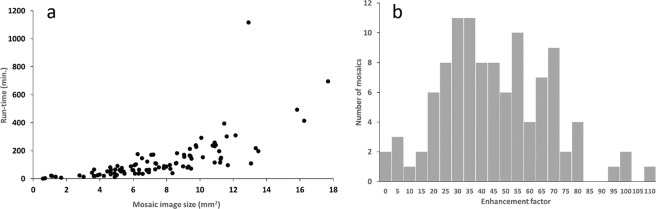
Characterization of mosaic computation and size. (**a**) Scatter plot for run-times for mosaic image generation in relation to mosaic image size. (**b**) Histogram of distribution of number of mosaics for each enhancement factor (enhancement factor is mosaic area divided by the area of a single 400 × 400 μm IVCM frame).

The average (mean ± standard deviation) percentage of SNP-classified images from a given eye was 59.2 ± 15.8%. The overall runtime of the mosaic image generation process is dominated by the image registration step, which exhibits a quadratic runtime behavior with respect to the SBNP-filtered dataset size when registering all possible image pairs. The following overall runtimes are therefore not normally distributed and are given as median (interquartile range). The overall runtime measured in the study process pipeline (i.e. not applying the runtime optimizations), was 88.6 (45.8, 166.1) minutes per eye (Fig. 1a). After employing the runtime optimizations, particularly including scaling down the images prior to phase correlation, the runtime of the entire process decreased to 3.0 (1.6, 5.7) minutes per eye. The resulting mosaic image quality was comparable with both approaches, as examined by visual comparison. Figure 1 shows a scatter plot of the runtime for mosaic generation in relation to the size of respective mosaic images in the study cohort, and to the number of individual raw IVCM images used to generate each corresponding mosaic. Runtimes are based on a Windows PC system (Core2 Duo, E8400, 2 × 3 GHz, 6GB RAM).

**Fig. 2:**
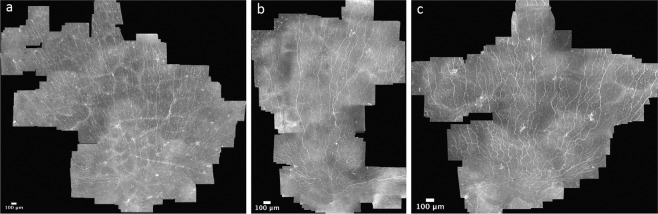
Representative IVCM mosaic images of the corneal subbasal nerve plexus for the three study groups. (**a**) Control. (**b**) Subject with PD with RLS. (**c**) Subject with PD without RLS.

The average (mean ± standard deviation) mosaic size per eye was 7.69 ± 3.53 mm² across a total of 106 mosaics, for the original process used for study evaluation. This corresponds to a mean enhancement factor of 48 across all 106 mosaics, meaning an equivalent mean tiled area of 48 individual IVCM image frames. Figure 1b shows the distribution of the number of mosaics with corresponding enhancement factor in the present dataset. The data presented here represents the mosaic with the largest size for each eye, differing slightly from prior analyses where in some cases average values from several mosaics per eye were used^30^. It is interesting to note that using the runtime-optimized algorithms, the average size of the largest mosaic image for a given eye decreased to 7.15 ± 3.30 mm², with either no area reduction or an area reduction of less than 5% of the original mosaic image area in 69% of the mosaics. It is worth noting that – for both the optimized and the non-optimized process alike – the original images that could not be integrated into the single largest mosaic image per eye are still assembled into separate, smaller mosaic images. Figure 2(a–c) shows representative SBNP mosaics from the control, PD + RLS and PD − RLS groups.

**Fig. 3:**
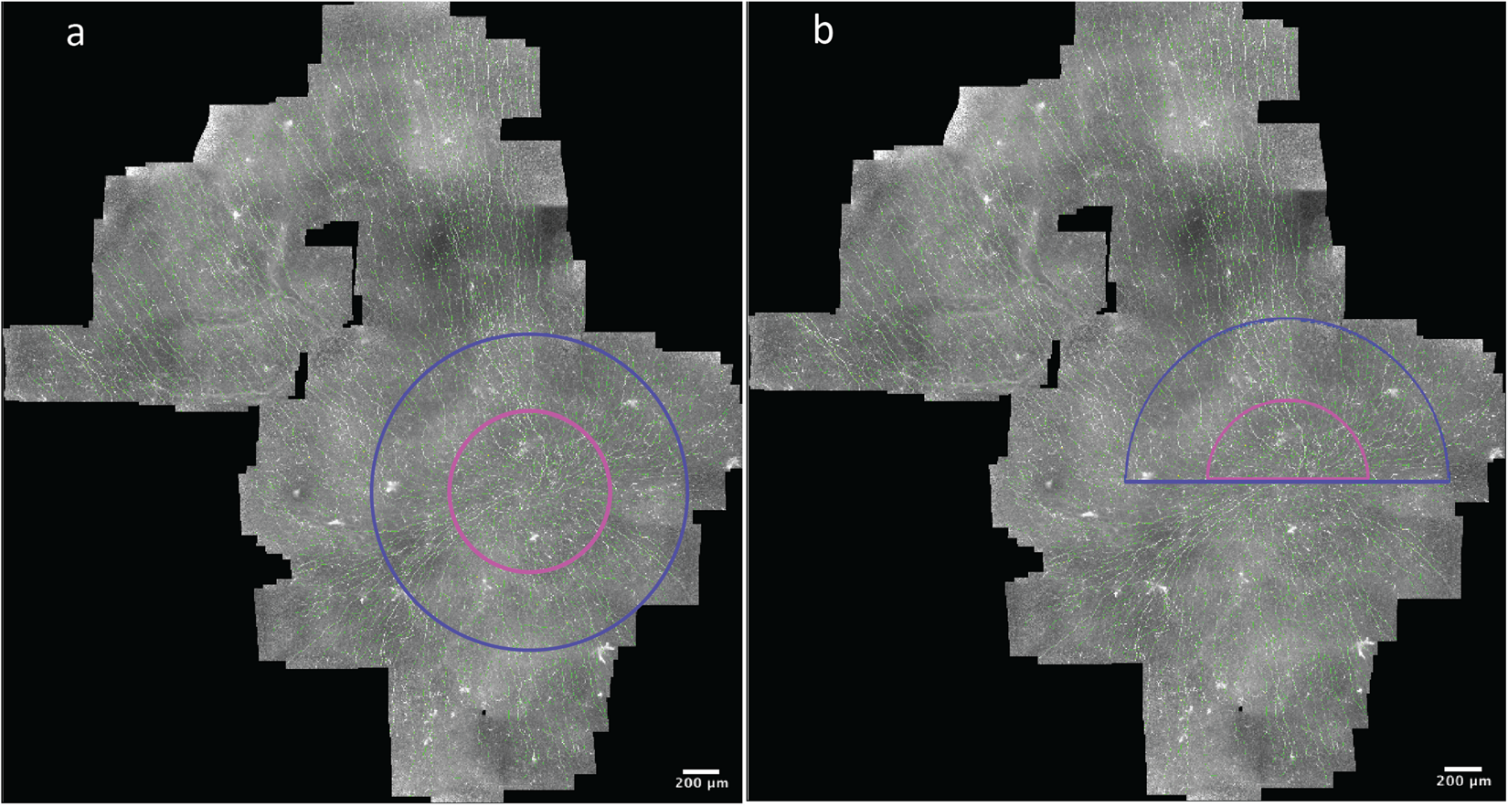
Mosaic image illustrating regions analyzed for inferocentral whorl parameter wCNFL. (**a**) Mosaic image depicting region of 800 μm radius (blue) and 400 μm radius (magenta) centered on the whorl center. (**b**) Mosaic image depicting upper half circle of 800 μm radius (blue) and 400 μm radius (magenta) based on the whorl center.

**Fig. 4:**
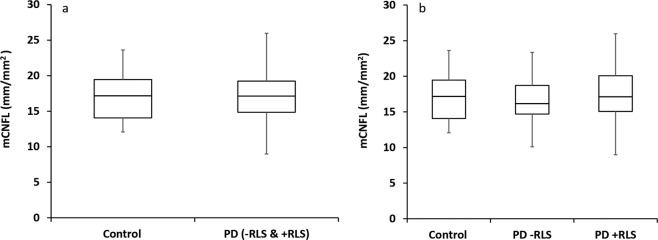
Box plots comparing mCNFL among the study groups. (**a**) Box plot comparing healthy controls vs. all Parkinson’s disease (PD) patients as a single group, t-test P = 0.62. (**b**) Box plot comparing all study subgroups including PD patients with and without RLS, ANOVA P = 0.79. Data represents quantification based on the single largest mosaic per eye, differing slightly from prior analyses where in some cases average values from several mosaics per eye were used^30^.

**Fig. 5:**
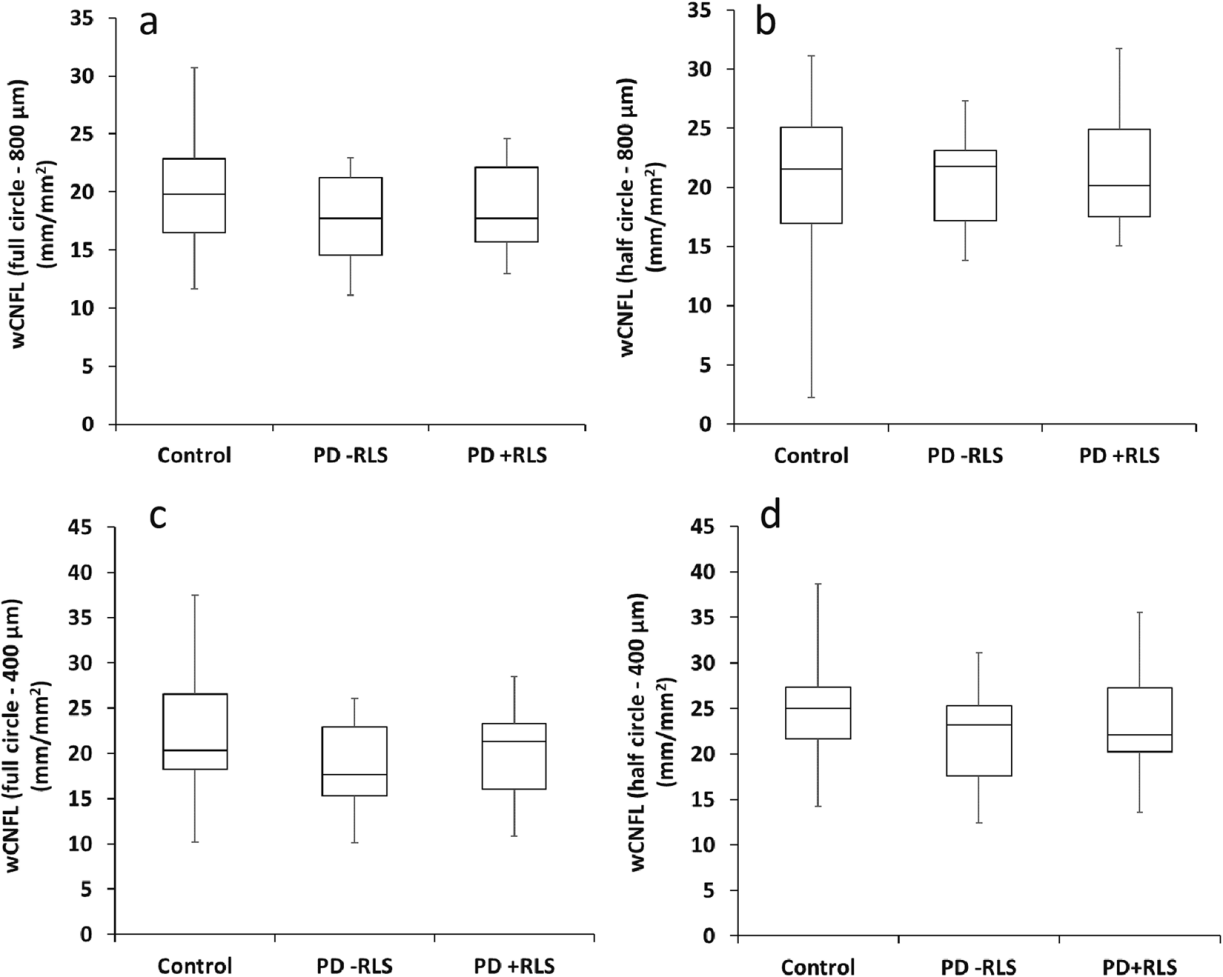
Box plots indicating comparisons of wCNFL across study groups. (**a**,**b**) Comparison of wCNFL across the three study groups. (**a**) Full circular whorl region of 800 μm diameter, ANOVA P = 0.47. (**b**) Half-circle region of 800 μm diameter, ANOVA P = 0.77. (**c**,**d**) The corresponding box plots for the whorl defined in a 400 μm diameter region. (**c**) Full circular whorl region of 400 μm diameter, ANOVA P = 0.36. (**d**) Half-circle region of 400 μm diameter, ANOVA P = 0.47.

Figure 3(a,b) depicts examples of the automatically traced mosaics. The traced nerves and identified branching points were quantified with respect to the entire mosaic image area, as well as the plexus area limited to the indicated whorl regions. We provide the nerve parameter data for these analyses as part of the current dataset. Example comparisons of mCNFL and wCNFL across the various groups are given in Fig. 4(a,b) and Fig. 5(a–d).

**Fig. 6:**
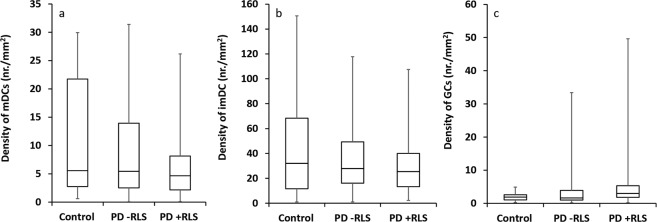
Box plots indicating comparisons for inflammatory cells across study groups. (**a**) Density of mature dendritic cells (mDCs), ANOVA P = 0.58. (**b**) Density of immature dendritic cells (imDCs), ANOVA P = 0.54. (**c**) Density of globular cells (GCs), ANOVA P = 0.19.

The validity of the manual inflammatory cell quantification in mosaics by the two observers was determined by the Bland-Altman analysis method of inter-observer agreement^53^. The mean, standard deviation (SD) and the 95% limits of agreement (LOA) between observers for the difference in cell density for each cell type across all mosaics is presented in Table 3, along with the correlation between observers measured by Pearson’s r. As an example of the inflammatory cell data, the density of mature and immature dendritic cells (mDCs, imDCs, respectively), and globular cells (GCs) are plotted across the three subject groups (Fig. 6a–c).

**Table 3:** Overview of inter-observer differences in dendritic cell quantification from mosaic images.

	Inflammatory cell type		
	mDCs	imDCs	GCs
Mean difference	9.3	−4.9	−0.8
SD	12.7	30.0	2.6
Lower 95% LOA	−15.6	−63.8	−5.9
Upper 95% LOA	34.3	54.0	4.3
Pearson’s r	0.68	0.74	0.98

Values represent the mean difference between the two observers, standard deviation of difference and lower and upper bounds of the 95% limits of agreement (LOA) for dendritic cell density in cells/mm^2^ of mosaic area, for each given cell type. Identification and quantification of inflammatory cells was based on three cell types: mDCs: Mature dendritic cells, imDCs: immature dendritic cells, and GCs: globular cells. The correlation coefficient between observers (Pearson’s r) is shown for each cell type.

## Data Availability

Computational codes used to, firstly, perform the depth-corrected mosaics synthesis used in the study and, secondly, for automated nerve tracing were developed by the academic institutions of Karlsruhe Institute of Technology and University of Padua, respectively, and are exclusively intended for scientific research use^30,31^. The developers of the respective algorithms are willing to apply the code to user-supplied raw IVCM data in the form of academic collaborations. Interested parties are requested to contact the respective researchers for mosaic creation (Allgeier) and automated nerve analyses (Scarpa).
